# The risk factors for postoperative temporary vocal cord paralysis after thyroid cancer surgery: an observational retrospective cohort study

**DOI:** 10.1097/JS9.0000000000001471

**Published:** 2024-04-19

**Authors:** Yujia Han, Yishen Zhao, Jiedong Kou, Jingting Li, Fang Li, Rui Du, Gianlorenzo Dionigi, Francesco Frattini, Nan Liang, Hui Sun

**Affiliations:** a Division of Thyroid Surgery, The China-Japan Union Hospital of Jilin University, Jilin Provincial Key Laboratory of Surgical Translational Medicine, Jilin Provincial Precision Medicine Laboratory of Molecular Biology and Translational Medicine on Differentiated Thyroid Carcinoma, Changchun City, Jilin Province, People’s Republic of China; b Division of General and Endocrine Surgery, Istituto Auxologico Italiano IRCCS, University of Milan, Milan, Italy

**Keywords:** cancer, influencing factors, thyroid surgery, vocal cord paralysis

## Abstract

**Objective:**

To analyze the potential factors influencing new-onset postoperative transient vocal cord paralysis (VCP) in thyroid cancer patients.

**Methods:**

Case information of 8340 thyroid cancer patients hospitalized at China-Japan Union Hospital of Jilin University, Jilin Province, China, in the Thyroid Surgery Department from January 2018 to December 2020 was collected retrospectively and analyzed. The possible influencing factors were analyzed using a *χ* ^2^ test, rank-sum test, and multiple logistic regression analysis. A nomogram was used to construct the clinical prediction model that was validated in the validation set by receiver operating characteristic, calibration curves, and Decision curve analysis.

**Results:**

The strengthening the reporting of cohort, cross-sectional, and case–control studies in surgery (STROCSS) guideline was followed to conduct a retrospective cohort study. A total of 8340 patients, including 1817 (21.8%) men and 6523 (78.2%) women, were enrolled in this study. The rate of temporary VCP was 3.6% (308/8340). Based on the results of postoperative laryngoscopy, the patients were divided into VCP group and non-VCP group. Comparative analysis between the groups revealed that potential factors associated with postoperative transient VCP were tumor location on the dorsal side of the gland (*P*=0.042), ultrasound showing a maximum nodal diameter >1 cm (*P*=0.002), multifocal carcinoma (*P*<0.001), invasion of surrounding tissue (*P*=0.005), lymph node metastases in the central compartment (*P*=0.034), lateral cervical lymph node metastasis (*P*<0.001), and prolonged operation (*P*<0.001). A multiple logistic regression analysis showed that the independent risk factors in postoperative transient VCP were T stage (OR=1.411, *P*=0.013, 95% CI: 1.075–1.853), multifocal carcinoma (OR=1.532, *P*=0.013, 95% CI: 1.095–2.144), and duration of surgery (OR=1.009, *P*<0.001, 95% CI: 1.006–1.012). Finally, a clinical prediction model was established via a nomogram and was validated in the validation set, although its diagnostic efficacy needs to be improved further.

**Conclusion:**

High T stage, multifocal carcinoma, and prolonged operation time may be independent risk factors for the occurrence of postoperative transient VCP in patients undergoing initial surgery for thyroid cancer.

## Introduction

Highlights

- The rate of temporary vocal cord paralysis was 3.6% (308/8340).
- High T-stage, multifocal carcinoma and longer operation time may be independent risk factors for the occurrence of postoperative transient VCP in patients undergoing initial thyroid surgery for thyroid cancer.
- A clinical prediction model was created using a nomogram and validated in the validation set, although the diagnostic efficacy of the model needs to be further improved.

*De novo* vocal cord paralysis (VCP) is a common complication after thyroid surgery and is associated with increased morbidity, mortality, prolonged hospitalization, and high costs^1–4^. It leads to impaired vocal cord function and atrophy, often manifested by symptoms such as altered speech, difficulty in pronunciation and swallowing, and shortness of breath^1^. Injury to recurrent laryngeal nerve (RLN) is a common cause of postoperative VCP, especially in patients who have undergone surgery for malignant thyroid tumors^2–4^. According to the literature, the incidence rate of permanent RLN injury is 0.5%^5^, while temporary RLN injury is 3%^6^. Temporary VCP occurs at a high rate of 2–13% within 2 weeks of thyroid surgery^7^. The factors influencing VCP after thyroid surgery are yet to be identified, requiring a large amount of data for evidence-based medicine. However, most of the literature^8–15^ has focused on the anatomical changes of the nerve itself and the effects of intraoperative procedures on the nerve, while only a few studies have assessed the underlying conditions of the patients and the biological characteristics of the tumor itself.

The early identification of cancer patients at risk for developing new-onset VCP may allow timely implementation of preventive and treatment strategies that could improve the early and late outcomes after thyroid surgery. This study aims to investigate the effects of patients’ baseline conditions, the biological characteristics of the tumor, and the intraoperative conditions on the occurrence of transient VCP after thyroid surgery to collect evidence for reducing the incidence of VCP after thyroid cancer surgery.

## Methods

### Study design, patients, setting, and time frame

A retrospective analysis of clinical information of patients who underwent thyroid surgery at the Department of Thyroid Surgery, China-Japan Union Hospital, Jilin University, Jilin Province, China, from January 2018 to December 2020 was conducted. The inclusion criteria were as follows: patients who underwent their first open thyroid surgery in our department and were confirmed to have thyroid cancer by pathological paraffin examination postoperatively. The exclusion criteria were as follows: patients with abnormal preoperative laryngoscopy, patients with previous neck surgery, and patients with incomplete case information. A total of 8340 patients were included in this study. In chronological order, a total of 6186 patients from January 2018 to December 2019 were included as a training set for investigating the factors affecting transient VCP after thyroid surgery and for establishing a prediction model. The remaining 2154 patients from January to December 2020 constituted the validation set for validation of the clinical prediction model. This study is reported according to STROCSS guidelines^16^.

### Ethics

The present study was approved by the Ethics Committee of China-Japan Union Hospital of Jilin University, and informed consent was obtained from all patients (approval number: 2023020719).

### Definitions

All patients underwent fiberoptic laryngoscopy on the first day after surgery to observe vocal cord movement. Vocal cord fixation, incomplete glottic closure, and unilateral or bilateral vocal cord dysfunction were diagnosed as VCP. These patients were followed up at 1 month, 3 months, and 6 months after surgery, including color Doppler ultrasound examination (including laryngeal vocal cords) and laryngoscopy. Recovery was defined as restoration of normal movement/mobility of vocal folds by laryngoscopic examination. If recovery was achieved before or at 6 months, then it would be temporary paralysis. BMI classification was based on international standards. The history of hypertension and hyperglycemia was self-reported by the patient. Family history was defined as the patient’s statement that a direct relative had thyroid cancer. The presence of a posterior nodule was defined as the presence of a nodule at the back of the thyroid gland as seen on preoperative ultrasound. The presence of a nodule near the trachea was defined as the presence of a thyroid nodule near the trachea as seen on the preoperative ultrasound. The maximum nodule diameter was defined as the maximum anterior-posterior diameter of the largest nodule measured on the preoperative ultrasound. Preoperative fine needle aspiration (FNA) of nodules was defined as performing FNA on suspicious malignant thyroid nodules before surgery. Preoperative lymph node FNA was defined as performing FNA on suspicious lymph nodes detected by preoperative ultrasound. The duration of surgery was defined as the time from the beginning to the end of surgery. Surgeons who performed ≥500 thyroid surgeries per year were defined as high-volume surgeons, and those who performed <500 thyroid surgeries per year were defined as low-volume surgeons.

### Statistical analysis

Categorical variables were presented as numbers and percentages, and continuous variables as medians (interquartile ranges). The comparisons between groups were performed using *χ* ^2^ test, Fisher’s exact test, or rank-sum test, with a two-sided *P*<0.05 considered as statistically significant. Multivariate logistic regression analysis was used to investigate the correlation between transient VCP and clinicopathological factors. Odds ratio with 95% CIs were calculated to determine the correlation among the potential predictive factors. All statistical analyses were performed using IBM SPSS version 23.0. The model was constructed and validated using Stata (version 12; Stata Corp, University Station). In order to construct the clinical prediction model in the form of a nomogram, variables were selected using the backward LR method. The receiver operating characteristic curve was used to evaluate the diagnostic performance of the model, and the calibration curve was used to represent its calibration.

## Results

### Basic characteristics and rates of VCP

This study retrospectively analyzed the case information from a total of 8340 patients who underwent initial thyroid cancer surgery between January 2018 and December 2020. Chronologically, 6186 patients from January 2018 to December 2019 were included in the training set, and the remaining 2154 patients from January to December 2020 comprised the validation set. As shown in Table 1, the cohort comprised 6523 (78.2%) female patients and 1817 (21.8%) male patients. 873/8340 (10.5%) had hypertension, 382 (4.6%) had diabetes, and 146 (1.8%) had a family history of thyroid cancer. Preoperative ultrasonography revealed that 2728/8340 (32.7%) patients had nodules on the dorsal side, while the other 1060 (12.7%) showed nodules near the trachea. The clinical characteristics of the training and validation sets were similar, and the rate of temporary VCP was 3.6% (308/8340).

**Table 1:** Patient characteristics

Variable	Total (N=8340)	Training set (N=6186)	Validation set (N=2154)	*P*	Variable	Total (N=8340)	Training set (N=6186)	Validation set (N=2154)	*P*
**Age (years), n (%)**					ST+CND+ULND	14 (0.2)	7 (0.1)	7 (0.3)	
＜55	7242 (86.8)	5379 (87)	1863 (86.5)	0.583	TT+CND+ULND	663 (7.9)	522 (8.4)	141 (6.5)	
≥55	1098 (13.2)	807 (13)	291 (13.5)		TT+CND+BLND	102 (1.2)	79 (1.3)	23 (1.1)	
**Sex,n (%)**					**Lymph node metastasis correlation**				
Female	6523 (78.2)	4841 (78.3)	1682 (78.1)	0.869	**CLNM, n (%)**				
Male	1817 (21.8)	1345 (21.7)	472 (21.9)		No	4678 (56.1)	3457 (55.9)	1221 (56.7)	0.519
**BMI, kg/m^2^ n (%)**					Yes	3662 (43.9)	2729 (44.1)	933 (43.3)	
＜18.5	216 (2.6)	169 (2.7)	47 (2.2)	0.118	**LLNM, n (%)**				
≥18.5 ＜25	4610 (55.3)	3440 (55.6)	1170 (54.3)		No	7482 (89.7)	5542 (89.6)	1940 (90.1)	0.531
≥25 ＜30	2898 (34.7)	2140 (34.6)	758 (35.2)		Yes	858 (10.3)	644 (10.4)	214 (9.9)	
≥30	616 (7.4)	437 (7.1)	179 (8.3)		**No. of CLNM**	0.0 [0.0-2.0]	0.0 [0.0-2.0]	0.0 [0.0-2.0]	0.342
**Basic Disease Information**					**No. of LLNM**	0.0 [0.0-0.0]	0.0 [0.0-0.0]	0.0 [0.0-0.0]	0.617
**Hypertension, n (%)**					**No. of LNM**	0.0 [0.0-2.0]	0.0 [0.0-2.0]	0.0 [0.0-2.0]	0.370
No	7467 (89.5)	5539 (89.5)	1928 (89.5)	0.966	**Combined thyroiditis, n (%)**				
Yes	873 (10.5)	647 (10.5)	226 (10.5)		No	6923 (83.0)	5147 (83.2)	1776 (82.5)	0.423
**Hyperglycaemia, n (%)**					Yes	1417 (17.0)	1039 (16.8)	378 (17.5)	
No	7958 (95.4)	5915 (95.6)	2043 (94.8)	0.140	**Pathological information of paraffin**				
Yes	382 (4.6)	271 (4.4)	111 (5.2)		**Maximum tumour diameter (cm), median [IOR]**	0.7 [0.4-1.0]	0.7 [0.4-1.0]	0.6 [0.4-1.0]	0.055
**Family history (%)**					**Multifocality, n (%)**				
No	8194 (98.2)	6083 (98.3)	2111 (98)	0.313	No	5006 (60)	3712 (60)	1294 (60.1)	0.956
Yes	146 (1.8)	103 (1.7)	43 (2)		Yes	3334 (40)	2474 (40)	860 (39.9)	
**Preoperative ultrasonic information**					**Invasion, n (%)**				
**Nodule located on the dorsal side, n (%)**					non-invasion	5943 (71.3)	4195 (67.8)	1748 (81.2)	＜0.001*
No	5612 (67.3)	3905 (63.1)	1707 (79.2)	＜0.001*	invasion of the capsule	1080 (12.9)	885 (14.3)	195 (9.1)	
Yes	2728 (32.7)	2281 (36.9)	447 (20.8)		invasion of the outer capsule	408 (4.9)	288 (4.7)	120 (5.6)	
**Nodule near the trachea, n (%)**					invasion of the surrounding tissue	909 (10.9)	818 (13.2)	91 (4.2)	
No	7280 (87.3)	5333 (86.2)	1947 (90.4)	＜0.001*	**Combined with nodular goiter, n (%)**				
Yes	1060 (12.7)	853 (13.8)	207 (9.6)		No	1703 (20.4)	980 (15.8)	723 (33.6)	＜0.001*
**Maximum nodule diameter, n (%)**					Yes	6637 (79.6)	5206 (84.2)	1431 (66.4)	
≤1cm	5321 (63.8)	3918 (63.3)	1403 (65.1)	0.135	**TNM, n (%)**				
＞1cm	3019 (36.2)	2268 (36.7)	751 (34.9)		**T stage**				
**Preoperative nodular FNA, n (%)**					T1+T2	8032 (96.3)	5961 (96.4)	2071 (96.1)	0.647
No	935 (11.2)	542 (8.8)	393 (18.2)	＜0.001*	T3+T4	308 (3.7)	225 (3.6)	83 (3.9)	
Yes	7405 (88.8)	5644 (91.2)	1761 (81.8)		**N stage**				
**Operation duration min, median [IOR]**	100 [80-130]	100 [80-135]	96 [75-125]	＜0.001*	N0	4584 (55.0)	3389 (54.8)	1195 (55.5)	0.796
**Operation scope, n (%)**					N1a	2905 (34.8)	2159 (34.9)	746 (34.9)	
UL+CND	4430 (53.1)	3261 (52.7)	1169 (54.3)	＜0.001*	N1b	851 (10.2)	638 (10.3)	638 (10.3)	
ST+CND	442 (5.3)	288 (4.7)	154 (7.1)		**M stage**				
TT+CND	2533 (30.4)	1932 (31.2)	601 (27.9)		M0	8323 (99.8)	6177 (99.9)	2146 (99.6)	0.045*
UL+CND+ULND	156 (1.9)	97 (1.6)	59 (2.7)		M1	17 (0.2)	9 (0.1)	8 (0.4)	

* *p*＜0.05.

BLND, bilateral lateral neck lymph node dissection; CLNM, central lymph nodes metastasis; CND,central lymph node dissection; LLNM lateral neck lymph nodes metastasis; LNM, lymph nodes metastasis; ST, sub-total thyroidectomy; TT, total thyroidectomy; UL, unilateral lobectomy; ULND, unilateral lateral neck lymph node dissection.

### Correlation analysis between postoperative temporary VCP and clinicopathological characteristics

To further analyze the potential factors influencing temporary VCP after thyroidectomy, patients were divided into VCP and non-VCP groups based on the laryngoscopy results on day one post-surgery. First, we compared the two groups to assess the correlation between transient VCP and clinicopathological features. As shown in Table 2, compared to the non-VCP group, the VCP group had a higher percentage of patients with hypertension (14.9 vs. 10.3%, *χ* ^2^=4.310, *P*=0.038), higher number of patients with tumors on the dorsal side (43.8 vs. 36.6%, *χ* ^2^=4.145, *P*=0.042), more patients with an ultrasound showing a maximum nodal diameter >1 cm (47.4 vs. 36.3%, *χ* ^2^=9.984, *P*=0.002), fewer patients who underwent preoperative FNA of the thyroid nodule (85.6 vs. 91.4%, *χ* ^2^=3.425, *P*=0.046), longer operation duration (130 min vs. 100 min, Z=7.245, *P*<0.001), larger maximum tumor diameter (0.8 cm vs. 0.7 cm Z=4.284, *P*<0.001), higher proportion of multifocal carcinomas (57.2 vs. 39.4%, *χ* ^2^=24.755, *P*<0.001), more patients with invasion of surrounding tissue (21.6 vs. 13.0%, *χ* ^2^=12.777, *P*=0.005), more patients with central lymph node metastasis (51.5 vs. 43.9%, *χ* ^2^=4.485, *P*=0.034), more patients with lateral cervical lymph node metastasis (22.2 vs. 10.0%, *χ* ^2^=29.669, *P*<0.001), higher total number of positive lymph nodes (1.0 vs. 0.0, Z=2.924, *P*=0.003), higher proportion of T4-stage in T-staging (6.7 vs. 0.4%, *χ* ^2^=120.095, *P*<0.001), higher proportion of N1b-stage in N-staging (22.2 vs. 9.9%, *χ* ^2^=30.673, *P*<0.001), and a higher proportion of operations with a greater extent than unilateral thyroid lobectomy (64.9 vs. 46.5%, *χ* ^2^=67.213, *P*<0.001). However, the incidence of VCP did not differ between patients operated on by high-volume surgeons or low-volume surgeons (data not shown). Thus, it can be deduced that these clinical characteristics are potential factors for temporary VCP after thyroidectomy.

**Table 2:** Univariate analysis of influencing factors of postoperative temporary VCP

	Non-VCP group (N=5992)	VCP group (N=194)	χ^2^/Z	*P*		Non-VCP group (N=5992)	VCP group (N=194)	χ^2^/Z	*P*
**Age (years), n (%)**					UL +CND+ ULND	93 (1.6)	4 (2.1)		
＜55	5213 (87.0)	166 (85.6)	0.340	0.560	ST +CND+ ULND	7 (0.1)	0 (0.0)		
≥55	779 (13.0)	28 (14.4)			TT +CND+ ULND	490 (8.2)	32 (16.5)		
**Sex,n (%)**					TT +CND+ BLND	67 (1.1)	12 (6.2)		
Female	4687 (78.2)	154 (79.4)	0.149	0.700	**Pathological information of paraffin**				
Male	1305 (21.8)	40 (20.6)			**Maximum tumour diameter, (cm), median [IOR]**	0.7 [0.4-1.0]	0.8 [0.5-1.3]	4.284	＜0.001*
**BMI, kg/m^2^ n (%)**	**Multifocality, n (%)**								
＜18.5	160 (2.7)	9 (4.6)	5.626	0.131	No	3629 (60.6)	83 (42.8)	24.755	＜0.001*
≥18.5 ＜25	3342 (55.8)	98 (50.5)			Yes	2363 (39.4)	111 (57.2)		
≥25 ＜30	2065 (34.5)	75 (38.7)			**Invasion, n (%)**				
≥30	425 (7.1)	12 (6.2)			non-invasion	4079 (68.1)	116 (59.8)	12.777	0.005*
**Basic Disease Information**					invasion of the capsule	857 (14.3)	28 (14.4)		
**Hypertension, n (%)**					invasion of the outer capsule	280 (4.7)	8 (4.1)		
No	5374 (89.7)	165 (85.1)	4.310	0.038*	invasion of the surrounding tissue	776 (13.0)	42 (21.6)		
Yes	618 (10.3)	29 (14.9)			**Lymph node metastasis information**				
**Hyperglycaemia, n (%)**					**CLNM, n (%)**				
No	5731 (95.6)	184 (94.8)	0.286	0.593	No	3363 (56.1)	94 (48.5)	4.485	0.034*
Yes	261 (4.4)	10 (5.2)			Yes	2629 (43.9)	100 (51.5)		
**Family history (%)**					**LLNM, n (%)**				
No	5895 (98.4)	188 (96.9)		0.140	No	5391 (90.0)	151 (77.8)	29.669	＜0.001*
Yes	97 (1.6)	6 (3.1)			Yes	601 (10.0)	43 (22.2)		
**Preoperative ultrasonic information**					**No. of CLNM**	0.0 [0.0-2.0]	1.0 [0.0-2.0]	1.875	0.061
**Nodule located on the dorsal side, n (%)**					**No. of LNM**	0.0 [0.0-2.0]	1.0 [-.0-3.0]	2.924	0.003*
No	3796 (63.4)	109 (56.2)	4.145	0.042*	**Case characteristics**				
Yes	2196 (36.6)	85 (43.8)			**Combined thyroiditis, n (%)**				
**Nodule near the trachea, n (%)**					No	4986 (83.2)	161 (83.0)	0.007	0.935
No	5173 (86.3)	160 (82.5)	2.352	0.125	Yes	1006 (16.8)	33 (17.0)		
Yes	819 (13.7)	34 (17.5)			**Combined with nodular goiter, n (%)**				
**Maximum nodule diameter, n (%)**					No	941 (15.7)	39 (20.1)	2.727	0.099
≤1cm	3816 (63.7)	102 (52.6)	9.984	0.002*	Yes	5051 (84.3)	155 (79.9)		
＞1cm	2176 (36.3)	92 (47.4)			**TNM, n (%)**				
**Preoperative FNA**					**T stage**				
**Preoperative nodular FNA, n (%)**					Tx	38 (0.6)	0 (0.0)	120.095	＜0.001*
No	514 (8.6)	28 (14.4)	8.058	0.005*	T1	5543 (92.6)	167 (86.1)		
Yes	5478 (91.4)	166 (85.6)			T2	204 (3.4)	9 (4.6)		
**Preoperative Lymph node FNA, n (%)**					T3	181 (3.0)	5 (2.6)		
No	5751 (96.0)	181 (93.3)	3.425	0.064*	T4	26 (0.4)	13 (6.7)		
Yes	241 (4.0)	13 (6.7)			**N stage**				
**Surgical information**					N0	3300 (55.1)	89 (45.9)	30.673	＜0.001*
**Operation duration min, median [IOR]**	100 [80-130]	130 [100-161]	7.245	＜0.001*	N1a	2097 (35.0)	62 (32.0)		
**Operation scope, n (%)**					N1b	595 (9.9)	43 (22.1)		
UL +CND	3193 (53.5)	68 (35.1)	67.213	＜0.001*	**M stage**				
ST +CND	280 (4.7)	8 (4.1)			M0	5983 (99.8)	194 (100.0)		1.000
TT +CND	1862 (31.1)	70 (36.1)			M1	9 (0.2)	0 (0.0)		

* *p*＜0.05.

BLND, bilateral lateral neck lymph node dissection; CLNM, central lymph nodes metastasis; CND,central lymph node dissection; LLNM lateral neck lymph nodes metastasis; LNM, lymph nodes metastasis; ST, sub-total thyroidectomy; TT, total thyroidectomy; UL, unilateral lobectomy; ULND, unilateral lateral neck lymph node dissection.

### Independent risk factors for transient postoperative VCP

To analyze the independent risk factors for transient postoperative VCP in thyroid surgery patients further, we input these factors in logistic regression analysis. As shown in Table 3, the potential independent influencing factors for postoperative transient VCP in thyroid surgery are T stage (OR=1.411, *P*=0.013, 95% CI: 1.075–1.853), multifocal carcinoma (OR=1.532, *P*=0.013, 95% CI: 1.095–2.144), and duration of surgery (OR=1.009, *P*<0.001, 95% CI: 1.006–1.012). These findings indicated that multifocality has a 1.5-fold higher risk of postoperative transient VCP compared to unifocality. In addition, the risk of developing VCP is 1.4 times higher in patients with a higher T-stage than in patients with a lower staging. These results suggested that high T-stage, multifocal carcinoma, and prolonged duration of surgery are independent risk factors for postoperative transient VCP in patients undergoing thyroid surgery.

**Table 3:** Multifactorial analysis of influencing factors of postoperative temporary VCP.

	B	S.E	Wald	OR (95% CI)	*P*
Operation duration (min)	0.009	0.002	30.583	1.009 (1.006–1.012)	＜0.001*
Multifocality	0.426	0.171	6.189	1.532 (1.095–2.144)	0.013*
T stage	0.344	0.139	6.157	1.411 (1.075–1.853)	0.013*

* *p*＜0.05.

### Development and validation of a clinical prediction model for postoperative transient VCP

**Figure 1:**
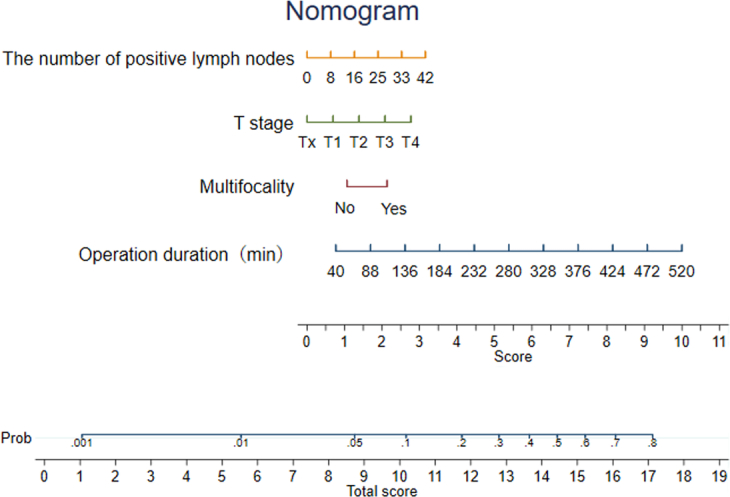
Nomogram for predicting postoperative temporary vocal cord paralysis.

**Figure 2:**
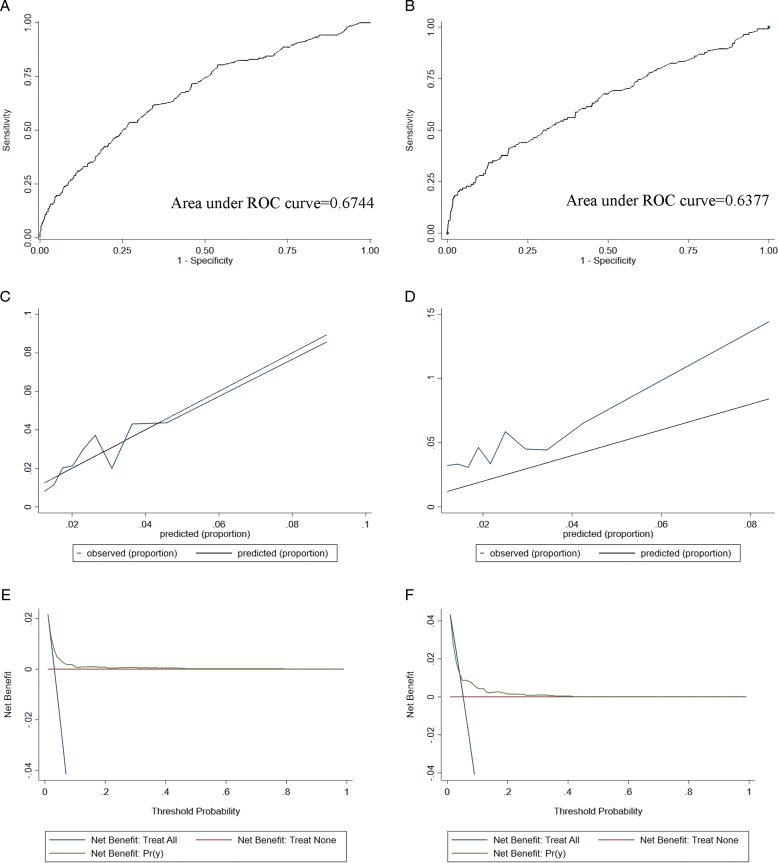
Verify the prediction model of postoperative temporary vocal cord paralysis, receiver-operating characteristics curve in the training set (A) and validation set (B) for the nomogram. Calibration curves of the nomogram in the training set (C) and validation sets (D). Decision curve analysis of the nomogram in the training set (E) and validation set (F).

Based on these risk factors, we established a clinical prediction model for postoperative transient VCP. First, we used a backward LR method with a significance level of *P*<0.05 to select the variables and the best model with the lowest AIC for model construction. The operation duration, T stage, cancer type, and total number of positive lymph nodes were included as variables in the model. Figure 1 illustrates the nomogram form of the model. In addition, the discriminative power of the model was validated by receiver operating characteristic analysis. As shown in Figure 2, the area under the curve values of the training and validation sets were 0.67 and 0.63, respectively. Consecutively, the calibration curve showed an agreement between the actual paralysis rate and the predicted probability, and the decision curve showed a net benefit of the model. These results suggested that we have attempted to build a prediction model for postoperative transient VCP in thyroid surgery, but its diagnostic performance needs to be further improved.

## Discussion

Postoperative temporary VCP is a common complication of thyroid surgery. This single center high-volume series of 8340 patients receiving thyroid surgery, showed low but still not negligible risks for postoperative morbidity, with temporary VCP occurring 3.6%. The current study suggested that transient VCP after thyroid surgery is related to several factors, however, the correlation among the factors is not yet clarified.

Several studies have proposed that the factors associated with postoperative transient VCP after thyroid surgery include intraoperative manipulations related to and the anatomical changes in RLN. For example, Staubitz *et al*.^17^ retrospectively analyzed 4598 patients from 82 centers; the results showed that intraoperative RLN injury is associated with an increased risk of VCP (OR=24.77). The study by Schneider *et al*.^18^ indicated that the most common mechanism of RLN injury during thyroidectomy is traction on the suspensory ligament of the thyroid gland, which is closely related to the anatomy of the nerve. Also, transection and thermal injury of the nerve during surgery can lead to severe RLN injury^19^. Some studies^20–23^ have also shown that variations in RLN are associated with an increased risk of postoperative VCP.

Nonetheless, only a few studies have examined the factors influencing transient VCP after thyroid surgery, focusing on patient demographics and the characteristics of the cancer. Some studies have shown the impact of patient demographics on postoperative VCP. For example, Wu *et al*.^24^ concluded that RLN diameter is positively correlated with age, weight, height, and BMI based on the analysis of 848 patients and 1357 RLNs. The RLN diameter was thinner in the RLN-injured group than in the non-injured group (1.71 mm vs. 1.55 mm, *P*=0.039). In addition, Ozemir *et al*.^25^ divided 111 cases into two groups based on the presence of diabetes mellitus and compared the latency and amplitude values of RLN monitoring results before and after thyroidectomy. The study found that diabetic patients had prolonged latency and decreased amplitude values, suggesting that the neurological changes in RLN are similar to those in peripheral nerves in diabetic patients. The increased values of latency after thyroidectomy suggested that RLN is susceptible to surgical trauma in diabetic patients.

Therefore, in the current study, we collected the baseline information of patients, such as age, sex, BMI, hypertension, hyperglycemia, and family history. The comparison between the groups revealed an association between hypertension and transient VCP after thyroid surgery. Additionally, thyroid cancer patients with a history of hypertension had a high risk of developing VCP post-surgery. This phenomenon has not been described previously, which might be because hypertensive patients have a rich vascular network in the posterior nerve, and small arterioles and venules are prone to hemorrhage during surgery, affecting the surgical field and increasing the difficulty of surgical dissection. However, since this is a retrospective study and the case data may be biased, further analyses in prospective studies are imperative to substantiate our findings.

The biological characteristics of thyroid cancer may influence temporary VCP after thyroid surgery, as reported previously. In a retrospective analysis of 197 cases, Mohammad *et al*.^26^ found that age, number of comorbidities, central lymph node metastasis, lateral lymph node metastasis, intraoperative extrathyroidal extension (ETE), length of stay(LOS), tumor size, and pathological ETE are potential risk factors for RLN injury based on univariate analysis. However, multivariate analysis revealed that only age (OR=4.8) and intraoperative ETE (OR=9.0) are independent factors influencing RLN injury. In the current study, we systematically evaluated the characteristics of the cancer, such as whether the nodules are located on the dorsal side, as indicated by preoperative ultrasound, whether there are nodules near the trachea, preoperative FNA, TNM staging, lymph node metastasis, and histopathological information. Univariate analysis demonstrated that hypertension, dorsally located nodes, maximum nodal diameter, preoperative thyroid nodal FNA, duration and extent of surgery, maximum tumor diameter, cancer type, extrathyroidal invasion, central compartment and lateral cervical lymph node metastases, the total number of positive lymph nodes, and T- and N-stages may be associated with temporary VCP after thyroid surgery. The multivariate regression analysis revealed that T-stage and multifocal cancer are independent risk factors for temporary VCP after surgery. Although the results of these variables are only available postoperatively, with the further development of preoperative diagnostic techniques, such as preoperative puncture cytology and biomarker identification. Therefore, the enhanced preoperative diagnosis of lymph node metastases in the future will have a substantial impact on the accurate prediction of VCP.

In analyzing the intraoperative factors, it was observed that patients in the VCP group underwent surgeries of longer duration compared to those in the non-VCP group. Furthermore, the duration of surgery was identified as an independent risk factor for temporary postoperative VCP. These findings align with the research conducted by Moreira *et al*.^27^ and Van *et al*.^28^. According to Van, after adjustment for confounding, only duration of surgery appeared to be a risk factor for any (temporary or permanent) RLN palsy: in total thyroidectomy, almost seven times higher risks were found among surgery durations >60 min (OR=6.70, 95% CI: 2.27–19.78); while 85% lower risks were found for hemithyroidectomies performed in <30 min (OR=0.15, 95% CI: 0.02–0.93); compared to 30–44 min as reference. The possible cause for this phenomenon can be attributed to several factors. Firstly, it is notable that patients who undergo lengthy surgical procedures often present with complex medical conditions, such as the presence of adhesions in cancerous regions, which necessitates the careful separation of tissues and the performance of a comprehensive lateral cervical lymph node dissection, involving a significant amount of surgical intervention. Consequently, the duration of the surgery is prolonged, consequently heightening the risk of temporary VCP. This implies that the surgeon should carefully contemplate the correlation between the duration of the procedure and the occurrence of postoperative VCP, and minimize the duration of surgery as much as possible. In this study, all surgeons standardized the intraoperative application of intraoperative neuromonitoring techniques for thyroid surgery. Therefore, the effect of whether or not the neuromonitoring technique was applied on postoperative transient VCP was excluded.

Several studies have assessed the intraoperative procedures during surgery. Herein, we focused on the characteristics of the cancer and concluded that in addition to the duration of surgery, the characteristics of the cancer, such as T stage and multifocality, are critical factors affecting temporary VCP. Nevertheless, the present study has some limitations. It is a retrospective study based on a large dataset from a single center, necessitating further validation by a multicenter prospective analysis. In addition, the diagnostic efficacy of the predictive model established in this study is satisfactory, although its performance needs to be optimized further. In our future study, we will incorporate more variables related to surgical procedures and expand the sample size to further enhance the diagnostic performance of the prediction model.

## Conclusion

In conclusion, this study focused on the characteristics of the patients and the features of the tumor. According to the statistical analysis and nomogram model, we identified high T-stage, multifocal carcinoma, and prolonged operation as independent risk factors for postoperative transient VCP in patients undergoing thyroid surgery. Although unmodifiable, these risk factors assist in the early identification of patients at risk, facilitating the timely implementation of prevention or treatment strategies. However, additional studies are essential to address whether the modification of these factors improves the early and late outcomes in patients undergoing thyroid surgery.

## Ethical approval

The present study was approved by the Ethics Committee of China-Japan Union Hospital of Jilin University, and informed consent was obtained from all patients (approval number: 2023020719).

## Consent

written informed consent was obtained from the patient for publication of this case report and accompanying images. A copy of the written consent is available for review by the Editor-in-Chief of this journal on request.

## Sources of funding

This study was sponsored by the Jilin Province Science and Technology Development Project [YDZJ202201ZYTS112]; the project of China-Japan Union Hospital [2023CL01]; the Project of Jilin Provincial Finance Department [2022SCZ09; 2023SCZ26; 2023SCZ51]. Science and Technology Research Project of Education Department of Jilin Province, China, [No.JJKH20221065KJ]; Jilin Province Health Research Talent Special Project [No.2020SCZ03]; Beijing Cihua Medical Development Foundation [J2023107004]. We also would like to thank many clinicians from our department for their support.

## Author contribution

N.L. and H.S.: conceptualization; N.L., YS.Z., and YJ.H.: methodology; N.L. and YJ.H.: formal analysis; YJ.H., JD.K., JT.l., F.L., and R.D.: investigation; N.L. and H.S: resources; Y.J.H: writing – original draft preparation; N.L., H.S., YJ.H., G.D., and F.F.: writing – review and editing; H.S.: supervision; N.L., Y.S.Z, and H.S.: project administration; N.L. Y.S.Z., and H.S.: funding acquisition. All authors have read and agreed to the published version of the manuscript.

## Conflicts of interest disclosure

The authors declared no conflicts of interest.

## Research registration unique identifying number (UIN)

The unique identifying number is: researchregistry9819. You will find your registration here: https://www.researchregistry.com/browse-the-registry#home/.

## Guarantor

All authors have read and agreed to the published version of the manuscript.

## Data availability statement

Please refer to the corresponding author when necessary.

## Provenance and peer review

This paper was not invited, externally peer-reviewed.
